# Proficiency in detecting vancomycin resistance in enterococci among clinical laboratories in Santiago, Chile.

**DOI:** 10.3201/eid0506.990623

**Published:** 1999

**Authors:** J A Labarca, L C McDonald, M E Pinto, E Palavecino, P González, E Cona, A Fernández, M S Giglio, W R Jarvis


the basis of the following characteristics: catalase negativity, α-hemolytic gram-positive cocci forming pairs and tetrads (not chains) in broth culture; growth in the presence of 40% bile and 6.5% NaCl and ability to hydrolyze esculin; pyrrolidony l-aminopeptidase positivity, leucine-aminopeptidase negativity; and production of acid from trehalose, sucrose, maltose, and lactose but not from sorbitol.

Susceptibility testing by the agar dilution method showed that the isolate was susceptible to penicillin-G (MIC = 0.12 µg/ml) and vancomycin (MIC = 0.25 µg/ml). On the basis of this case and previous reports (1,2), we believe that *A. viridans* is a potential pathogen that can cause serious infections in immunocompromised patients. The presumed route of infection in this patient was esophageal ulcers. Clinical microbiologists should pay close attention to α-hemolytic, catalase-negative streptococci recovered from sterile body sites that form tetrads rather than chains on Gram stain.

**Jafar H. Razeq,* Gloria M. Thomas,* and Daniel Alexander†**

*State of Maryland Department of Health and Mental Hygiene Laboratories Administration, Baltimore, Maryland, USA; †Franklin Square Hospital, Baltimore, Maryland, USA

### References

1. Swanson H, Cutts E, Lepow M. Penicillin-resistant *Aerococcus viridans* bacteremia in a child receiving prophylaxis for sickle-cell disease. Clin Infect Dis 1996;22:387-8.
2. Kern W, Vanek E. *Aerococcus* bacteremia associated with granulocytopenia. European Journal of Clinical Microbiology 1987;6:670-3.
3. Taylor PW, Trueblood MC. Septic arthritis due to *Aerococcus viridans*. J Rheumatol 1985;5:1004-5.
4. Untereker WJ, Hanna BA. Endocarditis and osteomyelitis caused by *Aerococcus viridans*. Mt Sinai J Med 1976;43:248-52.
5. Janosek J, Eckert J, Hudac A. *Aerococcus viridans* as a causative agent of infectious endocarditis. Journal of Hygiene, Epidemiology, Microbiology and Immunology 1980;1:92-6.
6. Pien FD, Wilson WR, Kunz K, Washington JA II. *Aerococcus viridans* endocarditis. Mayo Clin Proc 1984;59:47-8.
7. Kerbaugh MA, Evans JB. *Aerococcus viridans* in the hospital environment. Applied Microbiology 1968;16:519-23.

### Proficiency in Detecting Vancomycin Resistance in Enterococci among Clinical Laboratories in Santiago, Chile

**To the Editor:** Vancomycin-resistant enterococci (VRE) can be difficult to detect because of limitations in the susceptibility testing methods commonly used in clinical laboratories. Although VRE have not been reported in Chile, clinical isolates have been reported in Argentina (1) and Brazil (2). It is important to detect vancomycin resistance as early as possible, so infection control preventive measures can be instituted when they have their greatest impact. The microbiology laboratory is the first line of defense against VRE, as it plays a critical role in its recognition. In Chile, most laboratories follow the National Committee for Clinical Laboratory Standards recommendations for antimicrobial susceptibility testing and use disk-diffusion methods (3); however, these methods have limitations in detecting low levels of resistance to vancomycin in enterococci.

We evaluated the ability of referral microbiology laboratories in Chile to detect vancomycin resistance in five *Enteroccocus* spp. isolates with different susceptibility patterns for vancomycin, penicillin, and ampicillin. Of six referral laboratories that agreed to participate, four used the disk-diffusion method to evaluate antimicrobial susceptibility. Two used an agar dilution minimum inhibitory concentration (MIC) method, one as the only susceptibility testing method and the other in addition to disk diffusion. The participants correctly evaluated vancomycin susceptibility in 17 (57%) of 30 isolates.

The accuracy of detecting vancomycin resistance varied according to the level of resistance. Isolate 1, which had a high level of resistance (Van A phenotype, MIC 256 µg/ml), was evaluated correctly in 5 (83%) of 6 laboratories. Isolate 2, with a lower level of resistance (Van B, MIC 64 µg/ml), was evaluated correctly in 4 (67%) of 6 laboratories. Isolates 3 and 4, both with intermediate resistance (Van B, MIC 16-32 µg/ml, and Van C, MIC 8 µg/ml, respectively), were evaluated correctly by one laboratory each. Isolate 5 (vancomycin susceptible) was evaluated correctly by all laboratories. Susceptibility to penicillin and ampicillin was correctly identified in 53 (96.4%) of 55 isolates. Although laboratories correctly identified *E. faecium* and *E. faecalis* to the species level, most (4 of 5) did not correctly identify *E. gallinarum* (three misidentified it as *E. casseliflavus* and one as *E. faecalis*).

The results of this study are consistent with those of previous studies in the United States (4,5), South America (6), Spain (7), and Mexico (8). Although in countries like Chile, disk diffusion is practical and reliable for most susceptibility testing, detecting low-level vancomycin resistance in enterococci is difficult without supplementary testing. In Chile, as in other countries, strategies should be implemented to improve detection of these strains, including improvement of phenotypical and genotypical methods for VRE detection and species identification. Documentation of proficiency in detecting VRE is important for improving laboratory performance, detecting clinical isolates, and accurate and reliable reporting to local, national, and international surveillance systems.

**Jaime A. Labarca,* L. Clifford McDonald,† María Eugenia Pinto,‡ Elizabeth Palavecino,*‡ Patricia González,‡ Erna Cona,‡ Alejandra Fernández,‡ María Soledad Giglio,‡ and William R. Jarvis†**

*P. Universidad Católica de Chile, Santiago, Chile; †Centers for Disease Control and Prevention, Atlanta, Georgia, USA; and ‡Chilean Society for Infectious Diseases, Santiago, Chile

### References

1. Marin ME, Mera JR, Arduino RC, Correa AP, Coque TM, Stambulian D, et al. First report of vancomycin-resistant *Enterococcus faecium* isolated in Argentina. Clin Infect Dis 1998;26:235-6.
2. Cereda RF, Medeiros EA, Vinagre A, Rego ST, Hashimoto A, Febre N, et al. Epidemiologic analysis for acquisition of vancomycin-resistant enterococcus (VRE) in an intensive care unit in Brazil. In: Proceedings of the Eighth Annual Meeting of the Society for Healthcare Epidemiology of America; 1998; São Paulo, Brazil.
3. National Committee for Clinical Laboratory Standards (NCCLS). Performance standards for antimicrobial disk susceptibility tests: approved standard M2-A6, 6th ed. Villanova (PA): The Committee.
4. Tenover FC, Tokars J, Swenson J, Paul S, Spitalny K, Jarvis WR. Ability of clinical laboratories to detect antimicrobial agent-resistant enterococci. J Clin Microbiol 1993;31:1695-9.
5. Rosenberg J, Tenover FC, Wong J, Jarvis W, Vugia DJ. Are clinical laboratories in California accurately reporting vancomycin-resistant enterococci? J Clin Microbiol 1997;35:2526-30.
6. Cookson ST, Lopardo H, Marin M, Arduino R, Rial MJ, Altschuler M, et al. Study to determine the ability of clinical laboratories to detect antimicrobial-resistant *Enterococcus* spp. in Buenos Aires, Argentina. Diagn Microbiol Infect Dis 1997;29:107-9.
7. Alonso-Echanove J, Robles B, Jarvis WR. Proficiency of clinical laboratories in Spain in detecting vancomycin-resistant *Enterococcus spp*. The Spanish VRE Study Group. J Clin Microbiol 1999,37:2148-52.
8. McDonald LC, Garza LR, Jarvis WR. Proficiency of clinical laboratories in and near Monterrey, Mexico, to detect vancomycin-resistant enterococci. Emerg Infect Dis 1999;5:143-6.

### Food-Related Illness and Death in the United States

**To the Editor:** Dr. Mead and colleagues should be commended for attempting to estimate the prevalence of foodborne disease in the United States (1). Their study provides more complete estimates than previous studies in terms of the number of foodborne pathogens included; for example, it includes the first realistic estimate of the number of cases of disease due to Norwalk-like caliciviruses. However, the publication of these estimates raises some important issues.

Even though “accurate estimates of disease burden are the foundation of sound public health policy” (2), most of these estimates (in particular, the assumption that unknown agents are transmitted by food in the same proportion as known agents) were derived from assumptions rather than data. Known foodborne agents clearly cannot account for most gastrointestinal illnesses (1). However, illnesses from unknown agents may be as likely to have the transmission characteristics of rotavirus (1% foodborne) or *Cryptosporidium* (10% foodborne) as those of the Norwalk-like viruses (40% foodborne). Furthermore, it was assumed that detecting outbreaks or cases of toxin-mediated illnesses (e.g., due to *Bacillus cereus*, *Staphylococcus aureus*, or *Clostridium perfringens)* follows the model of *Salmonella*. In the authors’ entire list of known foodborne agents, data are presented for cases identified both from outbreaks and active surveillance for only three agents: *Salmonella, Shigella*, and *Campylobacter*. *Salmonella* is clearly the most highly characterized, hence the most attractive as a model. However, the ratios of the numbers of cases detected through active surveillance to the numbers of cases detected through outbreaks range from 10 for *Salmonella* to more than 400 for *Campylobacter*. What if the ratios for toxin-mediated illnesses were more

